# Effect of salinity on cable bacteria species composition and diversity

**DOI:** 10.1111/1462-2920.15484

**Published:** 2021-05-04

**Authors:** Ann‐Sofie Dam, Ian P. G. Marshall, Nils Risgaard‐Petersen, Laurine D. W. Burdorf, Ugo Marzocchi

**Affiliations:** ^1^ Center for Electromicrobiology, Section for Microbiology, Department of Biology Aarhus University Aarhus Denmark; ^2^ Section of Aquatic Biology, Department of Biology Aarhus University Aarhus Denmark; ^3^ Centre of Excellence for Microbial Systems Technology, Department of Biology University of Antwerp Wilrijk 2610 Belgium; ^4^ Center for Water Technology (WATEC), Department of Biology Aarhus University Aarhus Denmark

## Abstract

Cable bacteria (CB) are *Desulfobulbaceae* that couple sulphide oxidation to oxygen reduction over centimetre distances by mediating electric currents. Recently, it was suggested that the CB clade is composed of two genera, *Ca*. Electronema and *Ca*. Electrothrix, with distinct freshwater and marine habitats respectively. However, only a few studies have reported CB from freshwater sediment, making this distinction uncertain. Here, we report novel data to show that salinity is a controlling factor for the diversity and the species composition within CB populations. CB sampled from a freshwater site (salinity 0.3) grouped into *Ca*. Electronema and could not grow under brackish conditions (salinity 21), whereas CB from a brackish site (salinity 21) grouped into *Ca*. Electrothrix and decreased by 93% in activity under freshwater conditions. On a regional scale (Baltic Sea), salinity significantly influenced species richness and composition. However, other environmental factors, such as temperature and quantity and quality of organic matter were also important to explain the observed variation. A global survey of 16S rRNA gene amplicon sequencing revealed that the two genera did not co‐occur likely because of competitive exclusion and identified a possible third genus.

## Introduction

Cable bacteria (CB) are centimetres‐long, multicellular, filamentous *Desulfobulbaceae* (Pfeffer *et al*., 2012) that have been grouped into two genera: *Candidatus* Electrothrix and *Candidatus* Electronema (Trojan *et al*., 2016). CB's unique metabolism, referred to as electrogenic sulphide oxidation (e‐SOx; Malkin *et al*., 2014) allows them to couple the half‐redox reactions of sulphide oxidation and oxygen or nitrate reduction over centimetres distances by mediating electric currents (Nielsen *et al*., 2010; Marzocchi *et al*., 2014). Such peculiar metabolism significantly affects the benthic cycling of several elements including sulphur (Seitaj *et al*., 2017), nitrogen (Kessler *et al*., 2019; Marzocchi *et al*., 2020b, 2020a), iron (Risgaard‐Petersen *et al*., 2012), manganese (Sulu‐Gambari *et al*., 2016a) and phosphorus (Sulu‐Gambari *et al*., 2016b). Since their first discovery in sediment from Aarhus Bay (Denmark; Pfeffer *et al*., 2012), CB have been reported from a wide variety of environments, including brackish coastal and deep basins sediment (e.g., Klier *et al*., 2018; Marzocchi *et al*., 2018), salt marshes (e.g., Larsen *et al*., 2015;), coastal mud accumulation sites (van de Velde *et al*., 2016), bivalve reefs (Burdorf *et al*., 2017; Malkin *et al*., 2017), mangrove sediment (Burdorf *et al*., 2016), marine lakes (Malkin *et al*., 2014; Seitaj *et al*., 2017) and freshwater sites (Risgaard‐Petersen *et al*., 2015; Trojan *et al*., 2016; Kjeldsen *et al*., 2019). *Desulfobulbaceae* with the same electrogenic properties of CB have been found in aquifer sediments, although their 16S rRNA gene sequences were not more than 88% identical to *Ca*. Electrothrix and *Ca*. Electronema (Müller *et al*., 2016). Few studies however, have explored CB diversity at the sub‐family level i.e. genus (Klier *et al*., 2018) and species (Risgaard‐Petersen *et al*., 2015; Trojan *et al*., 2016; Marzocchi *et al*., 2018). Based on the analysis of existing 16S rRNA gene surveys, *Ca*. Electrothrix and *Ca*. Electronema have been suggested to have distinct marine and freshwater habitats respectively (Trojan *et al*., 2016). Despite this observation, the effect of salinity on CB diversity has not yet been systematically addressed. Moreover, the uncertainty about the overall diversity within the CB clade still limits our ability to discern the main factors controlling CB distribution and species composition.

In prokaryotic cells, the osmotic stress as induced by salinity is a driver for physiological adaptations (Walsh *et al*., 2013; Cabello‐Yeves and Rodriguez‐Valera, 2019) that, in turn, could lead to speciation. Freshwater and marine ecosystems are indeed inhabited by evolutionarily separated linages (Logares *et al*., 2009; Newton *et al*., 2011). The importance of salinity in determining the microbial community composition has been documented at the regional scale in transect studies across the Baltic Sea (Herlemann *et al*., 2011; Herlemann *et al*., 2016; Klier *et al*., 2018) and in estuary systems (Bouvier, 2002; Cottrell and Kirchman, 2003; Zhang *et al*., 2006). Although other parameters, including pH and quality of organic matter, may shape niche separation between freshwater and marine environments (Walsh *et al*., 2013), salinity has been identified as the major environmental determinant of microbial community composition at the global scale (Lozupone and Knight, 2007).

This study aims to determine the effect of salinity on CB species composition and diversity using two approaches: a salinity manipulation experiment and the analysis of datasets from previously published work both at the regional (Baltic Sea) and global scale. To test the tolerance of *Ca*. Electrothrix and *Ca*. Electronema to changes in salinity, we artificially altered the salinity of sediments that host CB and evaluated variations in e‐SOx activity and population structure. The effect of salinity on CB diversity and population structure at the regional scale was investigated by an in‐depth analysis of the dataset by Klier *et al*., 2018 on the benthic bacterial community along the salinity gradient of the Baltic Sea. Finally, we compiled and analysed previously published datasets based on 16S rRNA gene amplicon sequences available from the Sequence Read Archive (SRA) to evaluate the global diversity and species composition of CB across environments of different salinity.

## Results

### Sediment incubation at different salinities

To test the ability of the two genera to withstand shifts in salinity, sediment was collected in the proximity of Aarhus Harbour (56°08′17.7″N 10°12′44.8″E – Salinity 21), in a location known to host CB (Trojan *et al*., 2016), and from the hydrologically connected Brabrand Lake (56°08′25.7″N 10°08′41.0″E – Salinity 0.3). Sediment from each location was transferred to cores and incubated in parallel at salinity 21 and 0.3 for a month. Current density driven by e‐SOx, calculated from the conductivity of the sediment and the distribution of the electric potential (EP) as in Risgaard‐Petersen *et al*. (2014) showed that both sediment types hosted CB activity (Table 1) when incubated at native salinities. Harbour sediment incubated at lake salinity showed 93% reduction in current density compared to the native salinity. Lake sediment incubated at harbour salinity showed no CB activity. The 16S rRNA analysis indicated that only *Ca*. Electrothrix was present in the harbour sediment and only *Ca*. Electronema was present in the lake sediment (data not shown). Harbour sediment incubated at lake salinity showed a lower amount of unique CB ASVs (amplicon sequence variants) per sample and a drop in the relative abundance of the CB population within the community (−96%), compared to the native salinity (Table 1). To assess the variation in species composition within the CB population, the ASVs from the experiment and the dataset survey (see below) had been compared to 29 full length CB representative 16S rRNA sequences by using BLAST and subsequently ASVs > 97% identical to a reference sequence were grouped into operational taxonomic units (OTUs). These reference‐based‐OTUs will throughout the text be referred to as species, as they correspond to named candidate species where such names have been given (Trojan *et al*., 2016). In harbour sediment at native salinity the dominating species was *Ca*. Electrothrix communis constituting 69% of the CB population, with *Ca*. Electrothrix aarhusiensis the second most abundant, accounting for 17%. In harbour sediment incubated at lake salinity, *Ca*. Electrothrix communis and *Ca*. Electrothrix aarhusiensis accounted for 81% and 11% of the CB population respectively. Both described species of *Ca*. Electronema (i.e., palustris and nielsenii) were found in the lake sediment at native salinity. In lake sediment incubated at harbour salinity, a single core out of three contained one ASV identified as *Ca*. Electronema. This ASV had four reads in total, its relative abundance accounted for a marginal percentage (0.03) to the microbial community in the sample, and showed no activity during the incubations. Thus this could possibly represent a tag‐switching artefact during sequencing (Carlsen *et al*., 2012).

**Table 1:** Cable bacteria electrogenic activity (estimated as current density per square meter of sediment), number of unique amplicon sequence variances (ASVs) and percentage on the whole community in sediment from Aarhus Harbour and Lake Brabrand incubated for 4 weeks under native and altered salinities. Numbers represent mean ± SEM (*n* = 3).

	Salinity	Current density (mA m^−*2*^)	ASV (*n*. sample^−1^)	% community
Aarhus Harbour (Sal. 21)	21	88.9 (±2.3)	10.3 (±1.5)	3.5 (±0.26)
	0.3	6.0 (±0.18)	1.7 (±0.67)	0.12 (±0.02)
Brabrand Lake (Sal. 0.3)	21	0.0 (±0.00)	0.3 (±0.33)	0.01 (±0.01)
	0.3	14.7 (±0.28)	6.0 (±0.00)	1.5 (±0.61)

**Fig. 1:**
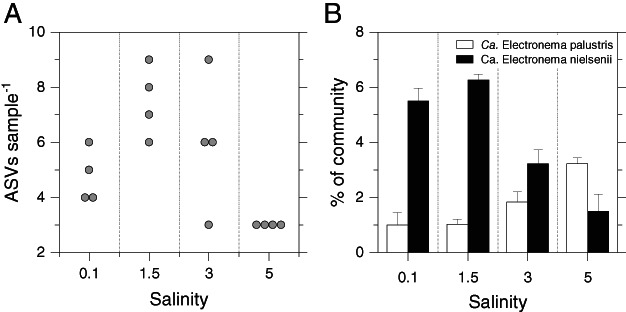
Beeswarm plot of the number of unique ASVs per sample (plot A), and bar plot of the relative abundance of reference‐based‐OTUs (*Ca*. Electronema; plot B) per sample of sediment from Brabrand Lake incubated at native (0.3) and altered (1.5, 3 and 5) salinity. Values are shown as mean ± SEM, *n* = 4).

To assess the tolerance of CB from the freshwater lake to a moderate increase in salinity, a second incubation was carried out where sediment from Brabrand Lake was incubated in a narrower salinity interval, *i.e*., 0.3, 1.5, 3 and 5. At the end of the incubation period, CB activity was comparable across all treatments (i.e., 5.3 ± 0.85 mA m^−2^, Av. ±SD, *n* = 4). The number of unique ASVs per sample was seen to change significantly through the salinity treatments (Fig. 1A; Kruskal–Wallis, *P* = 0.017) with the lowest number of unique ASVs at salinity 5. The 16S rRNA analysis indicated no significant effect of salinity on the relative abundance of CB in the community (Kruskal–Wallis, *P* = 0.08; Fig. 1B, cumulative of the two species). *Ca*. Electronema nielsenii and *Ca*. Electronema palustris were detected in all treatments (Fig. 1B). The ratio between the relative abundances of *Ca*. Electronema nielsenii and *Ca*. Electronema palustris changed significantly with the increasing salinity (Kruskal–Wallis, *P* = 0.007), with *Ca*. Electronema palustris becoming dominant at salinity 5.

### CB diversity and species composition in the salinity gradient of the Baltic Sea

**Fig. 2:**
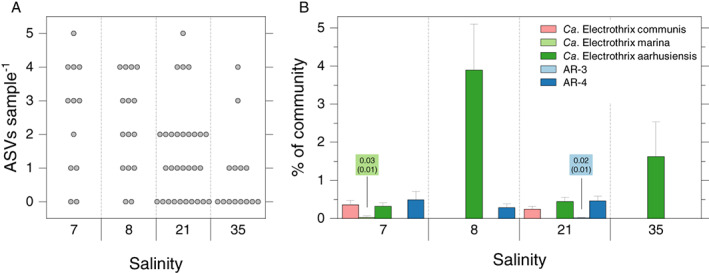
Plot A. Beeswarm plot of the number of unique ASVs per sample from the five different stations from the Klier *et al*., 2018 dataset. Plot B. Bar plot of the relative abundance of reference‐based‐OTUs (*Ca*. Electrothrix) from the Klier et al dataset sampled from five different stations in the Baltic Sea; *Ca*. Electrothrix communis, *Ca*. Electrothrix marina, *Ca*. Electrothrix aarhusiensis, AR‐3 AR‐4. Values are shown as mean ± SEM, *n* = 4. The number within boxes show values (as mean (SEM)) of the bars that are not clearly visible at the applied y‐scale. [Color figure can be viewed at wileyonlinelibrary.com]

**Fig. 3:**
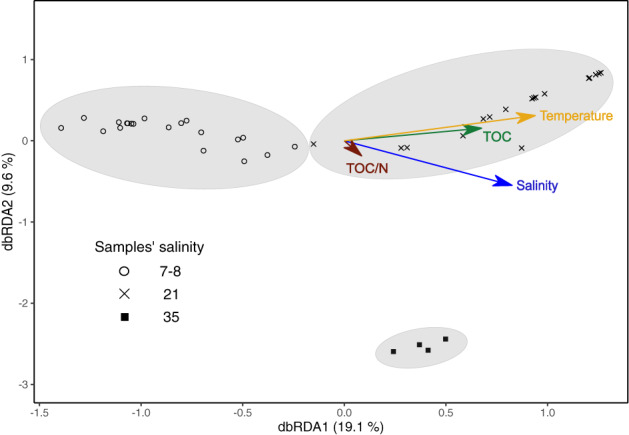
Redundancy analysis (RDA) of bray curtis values from samples of the Baltic Sea (Klier *et al*., 2018). Temperature (measured at sediment surface), TOC: total organic carbon of the sediment, TOC/N total organic carbon to nitrogen ratio of the sediment, Salinity (measured at sediment surface). [Color figure can be viewed at wileyonlinelibrary.com]

To assess the effect of salinity on CB diversity and species composition on a regional scale, we further investigated the benthic community 16S rRNA dataset compiled by Klier *et al*. (2018) in the Baltic Sea by specifically targeting the CB population. The dataset comprises samples from five stations with salinities of 4, 7, 8, 21 and 35. CB ASVs were detected at all stations, with the exception of the station at salinity 4. The number of unique CB ASVs found per sample differed significantly among stations (Kruskal‐Wallis, *P* = 0.01), with the lowest CB relative richness found at salinity 35 (Fig. 2A). *Ca*. Electrothrix aarhusiensis was present at all stations where CB were detected, being the dominant CB species at salinity 8, and the only species detected at salinity 35 (Fig. 2B). The other species were found in a narrower salinity interval. *Ca*. Electrothrix marina and AR‐3 were relatively rare and appeared only at salinity 7 (1 sample out of 9) and 21 (13 samples out of 21) respectively. *Ca*. Electronema was not present at any station. The highest number of different species was seen at salinity 7 and 21 where no clear dominance of specific species was observed. When salinity was grouped into intervals (low: 7 & 8; intermediate: 21; and high: 35) a dissimilarity analysis indicated significant differences of species composition among the intervals (PERMANOVA, *R* ^2^ = 21%, *P* = 0.0009). To analyse if the difference in species composition were consistent among all three salinity intervals and to test if other environmental factors available from the original dataset (i.e., temperature, total organic carbon, total sulphur, total nitrogen, total organic carbon/nitrogen ratio and porewater oxygen) could explain the apparent pattern in species composition, a distance‐based redundancy analysis (dbRDA) was performed. The potential predictors in this analysis were, however, highly correlated (Table S1) and, therefore, a variance inflation factor analysis was performed to select those that were only low to moderately correlated (variance inflation factor < 3). These were temperature and salinity of the overlying water, total organic carbon content of the sediment (TOC), and the quality of the organic carbon expressed as the TOC/N ratio. The dbRDA of Bray Curtis' values revealed that samples from sites with low, medium and high salinity grouped together in three distinct clusters (Fig. 3). The analysis further revealed that all four factors contributed to the cluster separation. Of these, temperature and salinity contributed the most as judged from the magnitude of the vectors in Fig. 3.

### CB diversity and species composition at the global scale

**Fig. 4:**
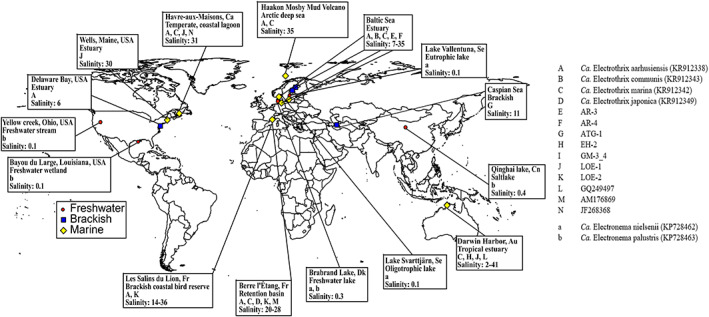
Cable bacteria distribution map generated from the reference‐based‐OTUs from the dataset survey. Red circles) freshwater environments, blue squares) brackish environments, yellow diamonds) marine environments. Letters indicate cable bacteria species defined by species name and/or accession number. Capital letters indicate *Ca*. Electrothrix and lowercase letters indicate *Ca*. Electronema species. [Color figure can be viewed at wileyonlinelibrary.com]

**Fig. 5:**
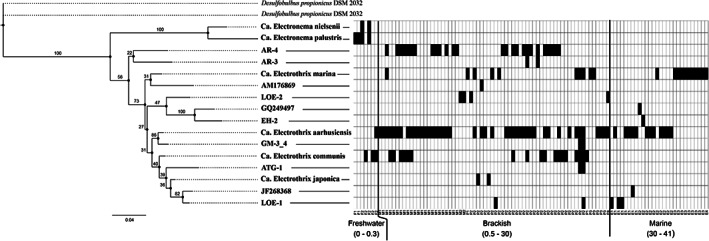
Phylogenetic tree of 16S rRNA sequences of cable bacteria from the global dataset survey. The tree of the was calculated by maximum likelihood (PhyML) with 100 bootstraps. The node numbers indicate the bootstrap values. Each column in the connected heat map is a sample with a specific salinity either containing (black) or not containing (grey) the species in the phylogenetic tree. The specific salinity in each sample can be seen in supplementary information (Table S4).

To assess the effect of salinity on the CB population at the global scale, we searched the SRA archive using IMNGS (Lagkouvardos *et al*., 2016) for 16S rRNA sequences with > 97% identity to 29 representative CB strains to find datasets containing CB from environmental samples of known salinity. The dataset survey generated OTUs across a broad variety of environments (Fig. 4 and Table S2). Samples from brackish environments (salinity 0.5–30, *n* = 66) had a significantly higher number of OTUs per sample (1.7 ± 1.0; av. ± SD) compared to marine (1.0 ± 0.2; salinity 30–41, *n* = 28) and freshwater environments (1.4 ± 0.5; salinity 0–0.5, *n* = 7; ANOVA, *p* = 0.002; Fig. 5). Similarly, the higher number of different OTUs were found at the brackish salinity interval (11 OTUs) compared to the freshwater (4 OTUs) and the marine (6 OTUs; Fig. 5) intervals. The CB clade based on these species divided into two distinct genus‐level branches, *i.e*., *Ca*. Electrothrix and *Ca*. Electronema (Fig. 5). In addition, the data revealed a possible third genus level branch containing two species (i.e., AR‐3 and AR‐4). These species were 92.5%–95.3% identical to the other species within the *Ca*. Electrothrix genus. The phylogenetic analysis of the 16S rRNA full‐length sequences with corresponding presence/absence data showed that *Ca*. Electronema were constrained to freshwater environments within a maximum salinity of 0.3. The remaining species showed no clear phylogenetic grouping with specific salinity preferences. Species belonging to *Ca*. Electrothrix were found across a broad range of salinity spanning between freshwater (min. salinity 0.1) and marine sites (max. salinity 41). Two species were detected below the freshwater threshold, but they were not exclusive to this environment. The majority of species (11 out of 14) were present within the brackish interval and seven of those were exclusively found within this salinity range. Three species were exclusively found in marine sediment. *Ca*. Electrothrix aarhusiensis was the most frequently found species. *Ca*. Electrothrix marina was the only species detected above salinity 35.

## Discussion

### The brackish‐marine and freshwater genera in the CB clade

Our global data survey showed that *Ca*. Electrothrix and *Ca*. Electronema had two distinct salinity preferences largely validating the previous hypothesis of a salinity split between the two genera (Risgaard‐Petersen *et al*., 2015; Trojan *et al*., 2016). Salinity has been reported as a key factor in shaping bacterial phylogenetic variation at different taxonomic levels (Crump *et al*., 1999; Logares *et al*., 2009; Campbell and Kirchman, 2013). The split between the two so far known CB genera appears to substantially comply with this pattern. However, although *Ca*. Electrothrix was preferentially found in brackish and marine environments, the two species aarhusiensis and communis hold the ability to live in freshwater sediments. On the contrary, *Ca*. Electronema appeared to be restricted to freshwater sites. Brackish sediment from Aarhus Harbour (salinity 21) incubated in freshwater (salinity 0.3) confirmed the results of the survey showing that *Ca*. Electrothrix could tolerate freshwater salinity, albeit with a severe reduction in activity (−93%) and relative abundance of their rRNA gene copies (−96%; Table 1). Salinity manipulation of freshwater sediment instead showed full inhibition of CB activity at salinity 21, possibly due to osmotic stress. Although *Ca*. Electronema was able to withstand changes in salinities of up to (at least) 5 under laboratory conditions, no *Ca*. Electronema species were detected at salinities higher than 0.3 in the global survey. This observation, in addition to the fact that the two genera were never found to co‐occur in the experiments and in the global dataset suggests an exclusive competition between *Ca*. Electronema and *Ca*. Electrothrix in the lower brackish interval.

Notably, our analysis revealed a possible novel third genus‐level branch that was detected in 28 samples across the 7–21 salinity interval. The two species within this branch were 92.5%–95.3% identical to the other species within the *Ca*. Electrothrix genus. This is only slightly above the conventional cut‐off (< 94.5%) for discriminating between different genera (Yarza *et al*., 2014) making the distinction uncertain. A more comprehensive, genome‐based analysis is needed to conclusively determine the status of this clade.

Whether the two CB genera evolved from a brackish/marine or a freshwater common ancestor is currently unknown. A compilation of the typical environment of the species within the *Desulfobulbaceae* family (Table S3) revealed that the majority of species (23 out of 28) were found at salinities within the brackish‐marine interval, with the remaining five species isolated from environments with an unspecified or freshwater salinity. *Desulfobulbaceae* is thus mainly a brackish‐marine family with a few cases of freshwater species adapted from their marine ancestors. This suggests a marine origin of the CB clade and thus that *Ca*. Electronema lost the ability to tolerate higher salinities. A marine origin of the genus *Ca*. Electrothrix has also been proposed by Klier *et al*. (2018) based upon marine terms from the *EnvO* (Buttigieg *et al*., 2016) analysis and a higher abundance in marine salinities. Recently, it was found that the freshwater lineage in SAR11 (Pelagibacterales) had a smaller genome than its brackish and marine counterparts and that genes encoding osmolyte uptake proteins were absent, indicating that these genes were lost upon speciation (Henson *et al*., 2018). *Ca*. Electronema was similarly found to have a genome of about 1 Mbp smaller than *Ca*. Electrothrix (Kjeldsen *et al*., 2019) indicating a possible loss of genes for osmoregulation.

### CB diversity is influenced by salinity

Results from the sediment incubations indicate a typical higher number of unique ASVs per sample within the *Ca*. Electrothrix genus compared to *Ca*. Electronema. This pattern was confirmed in the dataset survey. The broader species richness within the genus *Ca*. Electrothrix could simply reflect the broader salinity range (i.e., availability of ecological niches) encompassed by the brackish/marine interval compared to the freshwater one. An additional explanation may include an accelerated molecular evolution (i.e., the amount of evolutionary diversification, Logares *et al*., 2009) as driven by osmotic stress in the brackish and marine salinity interval. Logares *et al*. (2010) observed that the molecular evolution was 7.5 times lower within the SAR11 freshwater cluster compared to the marine and brackish clusters and proposed that the increase in diversity is linked to the negative effects of salt in the fidelity of DNA replication as seen for freshwater and halophilic crustaceans (Hebert *et al*., 2002). Such different evolutionary ‘pace’ could possibly contribute to explain the lower species richness in *Ca*. Electronema compared to *Ca*. Electrothrix observed in our study.

Salinity influences the relative richness also within the two genera. For *Ca*. Electronema we observed a variation in number of unique ASVs per sample across the salinity interval 0.3–5 (Fig. 1) with lowest numbers at salinity 5. For *Ca*. Electrothrix in the global dataset, the number of species was significantly higher at the brackish salinity interval (0.5–30) compared to the freshwater (0.1–0.3) and marine interval (30–41). This indicates optima with higher species richness for both genera in the mid‐area of their respective salinity ranges.

### Salinity is one factor controlling CB species composition

The PERMANOVA analysis showed significant differences in species composition among salinity intervals in the Baltic Sea dataset and dbRDA analysis suggested that the salinity of the overlying water was a major driver of this pattern (but not the only one). Although a salinity‐driven phylogenetic relationship below genus level was not found in the global dataset survey, some species within *Ca*. Electrothrix showed preferences for a specific range of salinities (Fig. 5). Such preferences are expected to contribute to the alteration of the species composition in environments of different salinity. Whereas *Ca*. Electrothrix aarhusiensis was found across the entire salinity spectrum, showing itself to be a salinity‐tolerant species, *Ca*. Electrothrix communis was only found at low and intermediate salinities, and *Ca*. Electrothrix marina was the only species found at the highest salinities (Fig. 5). Despite some species‐specific preferences/adaptations to low or high salinity, in general, brackish waters appear to sustain higher numbers of species with up to six species co‐occurring at the same site. A lower diversity at the community level has been reported from microbial mats at marine salinities compared to brackish‐water by Bolhuis *et al*. (2013) although other co‐vary factors might have contribute in determining this pattern. Generally Deltaproteobacteria, to which CB belong, have been found to have a high abundance in brackish habitats (Edmonds *et al*., 2009; Pavloudi *et al*., 2016, 2017).

Besides controlling patterns of presence/absence, variations in salinity appeared to modify the relative abundance of co‐occurring species within the CB population. In the manipulation experiment, reducing the salinity from 21 to 0.3 caused a decrease in the relative abundance of *Ca*. Electrothrix aarhusiensis from 17% to 11%, and a simultaneous increase of the relative abundance of *Ca*. Electrothrix communis from 69% to 81%. Accordingly, quantification of CB densities via Fluorescence *in situ* Hybridization of Baltic Sea sediment showed an inversion of the dominance pattern between these two species with *Ca*. Electrothrix communis dominating at salinity 7.3 and *Ca*. Electrothrix aarhusiensis dominating at sites exposed to inflow of salty waters (salinity 13–14; Marzocchi *et al*., 2018). Such alteration of the relative proportion between species is suggestive of a salinity control on the CB species fitness, possibly influencing dominance relationships.

Notably, *Ca*. Electrothrix communis was found exclusively in the Baltic Sea (dataset by Klier *et al*., 2018 and Aarhus Harbour samples) while *Ca*. Electrothrix aarhusiensis was found in a wide variety of environments (Figs. 4 and 5). In the Baltic Sea, the relative abundance of *Ca*. Electrothrix aarhusiensis was highest when *Ca*. Electrothrix communis was not present. Thus, whereas *Ca*. Electrothrix aarhusiensis appears to be a generalist species, *Ca*. Electrothrix communis could be better adapted to the specific environmental conditions found in the Baltic Sea and in the connected Aarhus Harbour.

Similarly, to *Ca*. Electrothrix, the *Ca*. Electronema species from Brabrand Lake displayed specific salinity patterns with *Ca*. Electronema nielsenii dominating at salinities from 0.3 to 3 and *Ca*. Electronema palustris dominating at salinity 5. These salinity preferences could influence not only the species composition but also the distribution of the two species. Accordingly, *Ca*. Electronema nielsenii was exclusively found at freshwater sites (Fig. 5), whereas *Ca*. Electronema palustris was also detected in a ‘saltlake with high freshwater inflow’ where the ability to withstand transient increase in salinity might be essential.

Our data show that salinity is an important although not the only factor in determining species composition within the CB population. In the Baltic Sea dataset, the seemingly tolerant *Ca*. Electrothrix marina could not, for instance, be found at salinity 35. Furthermore, stations that differed only marginally with regard to salinity (i.e., 7 to 8) showed substantially different species composition (Fig. 2). Temperature, TOC and TOC/N were also found to be controlling factors for the species composition within the CB population. Remarkably, no CB were found at salinity 4 in the Baltic Sea dataset despite our laboratory experiments with sediment from the Baltic Sea (Aarhus Harbour) or Baltic Sea catchment (Brabrand Lake) show that species from both genera are able to withstand such salinity. The overall abundance of CB in Baltic Sea sediments has been proposed to depend upon the presence of an active sulphur cycle and being related to the availability of oxygen in the bottom water (Hermans *et al*., 2019). With respect to oxygen, higher densities were found in basins that experience seasonally oxic conditions, and low densities and absence reported from permanently oxic and anoxic sites respectively (Marzocchi *et al*., 2018; Hermans *et al*., 2019). Within transiently oxic sites, however, CB may vary from being undetectable to appear at high densities possibly depending on temporal dynamics and the concurrent establishment of other microorganisms that can compete for the same geochemical niche *i.e*. Beggiatoaceae (Seitaj *et al*., 2015; Yücel *et al*., 2017; Hermans *et al*., 2019). Resource availability and competition relationships might thus have contributed to determine the absence of CB at the station at salinity 4 in the Baltic Sea dataset.

Further studies involving larger datasets or manipulative experiment will enable to discern the relative importance and potential interplay among these factors in controlling presence/absence and diversity patterns of CB species.

## Conclusion

This study supports the general distinction into a freshwater and brackish/marine genus. Although the two genera may share a common niche in the upper freshwater/low brackish water interval, they were never found to co‐occur likely because of competitive exclusion. Salinity is a strong indicator of the distribution of CB on a genus and to some extent, to the sub‐genus level. Furthermore, salinity was found to significantly influence both the species composition and the diversity within each genus. The brackish/marine genus *Ca*. Electrothrix was found to be more diverse than the freshwater genus *Ca*. Electronema. The species richness of CB within each genus appeared to be influenced by salinity with optima in the mid‐area of their respective salinity intervals. Both genera had species displaying specific salinity preferences, which in turn influenced the species composition at different salinities. However, other factors such as temperature and the quantity and quality of the organic matter of the sediment also influence the species composition.

## Experimental procedures

### Site, sample collection and incubation

Freshwater sediment was collected in February 2018 (first incubation) and February 2019 (second incubation) at the eastern end of Brabrand Lake in Aarhus, Denmark (56°08′25.7″N 10°08′41.0″E). The salinity of the bottom water (0.3) was recorded via a conductivity meter (SG3, Mettler Toledo, China). In February 2018, sediment was also collected at Marselisborg Marina in Aarhus Harbour (56°08′17.7″N 10°12′44.8″E, 26/02/2018). Salinity of the bottom water was 21. At both sites, about 30 cm of surface sediment was collected by sediment corer at two meters water depth. After collection, the sediment was homogenized and sieved (mesh size: 5 mm) to remove in fauna, debris and rocks that might damage microsensors during later measurements. These pre‐treatment procedures are known to inhibit any e‐SOx possibly active at the time of sampling, however the CB population can re‐establish and reach maximum densities/activities within 2–4 weeks after exposure of the sediment to aerated water (Schauer *et al*., 2014; Marzocchi *et al*., 2020b).

In the first experiment (February 2018), the harbour and lake sediments were incubated at native (i.e., 21 and 0.3 respectively) and inverted salinities (i.e., 0.3 and 21 respectively). In a second experiment (February 2019), the lake sediment was incubated at native (0.3) salinity and at salinity 1.5, 3 and 5. Waters at different salinity were prepared by mixing tap water with Red Sea Salt (Red Sea Fish Pham, Eilat, Israel).

After sieving, the sediment was suspended in the saltwater treatment to reach a consistent salinity throughout the sediment. Water was made anoxic via N_2_ flushing prior to mixing it with sediment to avoid loss of reduced Fe and S species. The sediment was allowed to settle through the water (1 day) before being transferred to 3–4 cores (diameter: 4.5 cm, length: 6.5 cm) per salinity treatment. Cores were then transferred to 4 L aquariums containing water with the specific salinity treatment. Air pumps ensured oxic conditions in the aquariums. The sediment cores were incubated for 1 month.

### Assessment of CB activity by EP measurement and calculation of e‐SOx activity

The principle of EP sediment profiling and its application to quantify CB activity has been described in great detail in Damgaard *et al*. (2014) and in Risgaard‐Petersen *et al*. (2014). Briefly, spatial segregated redox half‐reactions as driven by the metabolic activity of CB induce electric fields in the sediment that can be measured as an increase in EP with depth. The current density *J* (current per unit of sediment area, in mA m^−2^) mediated by CB can be calculated by the electric field *E* and the sediment conductivity *σ* by means of Ohm's law (Risgaard‐Petersen *et al*., 2014):

Je=σ*E

Sediment conductivity was estimated from overlying water conductivity corrected for porosity and tortuosity (Archie, 1942). The conductivity of the overlying water *σw* was measured by a conductivity meter (Mettler Toledo, Fisher Scientific, USA). The electric field E was determined from the variation of the EP with depth in the linear range of the depth profile. EP depth profiles were conducted using a EP microsensor (Damgaard *et al*., 2014) as described in (Marzocchi *et al*., 2018) at depth intervals of 200 μm.

### 16S rRNA extraction, PCR and sequencing

Cut‐off syringes of 3 ml were used to sample the upper 2.5 cm layer of the sediment in each core. Samples were then snap frozen in liquid nitrogen and stored at −80°C. The 16S rRNA was extracted with the RNA PowerSoil® Total RNA Isolation Kit (Qiagen) and subsequently DNase treated with the TURBO DNA‐free™ Kit (Ambion, Applied Biosystems) to remove potential DNA contamination. DNA contamination was checked with qPCR. The extracted 16S rRNA was amplified by reverse transcription PCR (RT‐PCR) using the OneStep RT‐PCR kit (Qiagen) which yielded the complementary DNA (cDNA). The primers added were Bac341F and Bac805R (Herlemann *et al*., 2011) to amplify the hypervariable region V3‐V4 of the 16S rRNA gene. Additionally, a DNA contamination control was run using HotStarTaq Master Mix Kit (Qiagen). The sequencing libraries of the V3‐V4 region were prepared according to the Illumina MiSeq system instructions (Illumina, 2013). The cDNA products were amended forward and reverse adapter overhangs for the adapter PCR by use of the KAPA Hifi Hotstart Readymix (KAPA Biosystems). Lastly, index PCR added indices by use of the Illumina Nextera XT Index Kit. After each PCR run the PCR products was purified with AMPure XP beads (Beckman Coulter) and checked by agarose gel electrophoresis (1.5% wt/vol). To measure the concentration of the DNA products a Qubit 2.0 Fluorometer was used (Thermo Fisher) after which the final products were sequenced (Miseq., Illumina). All PCR details are specified in the supplementary information section (Table S5–10). Sequences are available in the NCBI/SRA database under accession number PRJNA702292.

### Generation of the cable bacteria global data set

The IMNGS tool (Lagkouvardos *et al*., 2016) was used to find datasets for this study, with 10 16S rRNA gene sequences representative for CB used as query sequences in the ‘similarity query’ function, with the 97% identity cut‐off. Coordinates from the datasets were extracted to locate the respective studies on a world map. The datasets with freshwater, brackish and marine origin were then chosen from this map. The raw fastq files from the datasets were downloaded from NCBI via the SRA toolkit version 2.9.2. Subsequently, the primers from the raw files were cut using cutadapt (Martin, 2011). If the fastq files were larger than 4 MB, ShortRead version 1.46.0 (Morgan *et al*., 2009) was used to take a random subsample (10,000 reads) of the dataset before running the data through the DADA2 (Callahan *et al*., 2016) protocol. This ensured a feasible processing time.

### Sequence data, phylogenetic and statistical analyses

The open‐source software package DADA2 version 1.12 (Callahan *et al*., 2016) was used for error correcting and modelling of the sequenced data from both the experiment and global dataset survey. For datasets where comparisons of richness were made, reads were subsampled to an identical number of reads per sample using the ShortRead package (Morgan *et al*., 2009). This applied to the datasets from Klier *et al*. (2018) (subsample: 3000 reads/sample) and for the second incubation of sediment from Brabrand Lake (subsample: 29900 reads/sample) to normalize the dataset for accurate richness comparisons (Figs. 1A and 2A).

In the DADA2 pipeline the function *filterAndTrim* was used with the following arguments; the forward and reverse sequences were trimmed to around 220 base pairs (bp) based on the quality profiles. In some datasets only forward sequences were available. Furthermore, the filtering parameters were *maxN =* 0, *maxEE =* c(2,2) and *truncQ =* 2. These values were chosen based on the online DADA2 standard operating procedure (benjjneb.github.io/dada2/tutorial). The function *removeBimeraDenovo* was used to remove chimeric sequences. For classification the Silva taxonomic training data formatted for DADA2 was used (Silva version 132, Quast *et al*., 2013). All 16S rRNA gene sequences from the dataset survey and from the experiment were compared to a reference dataset consisting of 29 CB full‐length 16S rRNA sequences using BLAST (Boratyn *et al*., 2013; ncbi BLAST version 2.9.0). Only sequences from our dataset that were > 97% identical to sequences in the reference dataset were used for generating a reference‐based‐OTU table. The reference sequences used to make this table were aligned *de novo* and manually inspected using the software tool Geneious version 11.1.5 (https://www.geneious.com/). Geneious was then used for generating a phylogenetic tree based on these aligned sequences. The phylogenetic tree was calculated by maximum likelihood (PhyML 3.0, Guindon *et al*., 2010) with 100 bootstraps.

The Kruskal‐Wallis chi‐square test were used to test whether the number of ASVs differed between salinity intervals in the data set survey and for the Baltic data set alone. This nonparametric test was chosen as the data was not normally distributed and thus violated the assumptions of the analysis of variance. For the Baltic data set a permutational multivariate analysis of variance (PERMANOVA) was conducted in R version 3.5.1. The analysis was based on a dissimilarity index matrix of the samples containing CB from the four different sites in the Baltic generated by the R package ‘vegan’ version 2.5–4 by use of the function *vegdist* followed by *adonis2* (Oksanen *et al*. 2019). The dissimilarity index used was Bray Curtis. This similarity index matrix was used for generating a constrained ordination by a distance‐based redundancy analysis (dbRDA) by use of the function *dbrda* in the ‘vegan’ package as well. An initial dbRDA was performed including all available environmental factors. A variance inflation factors (VIF) test was performed on this initial model. This was done to assess a possible inflation of variance explained by these factors due to correlation between them. The test was performed by the function *vif.cca* in the ‘vegan’ package. After each VIF test the factor with a VIF values > 3 was excluded from the model. This was done repeatedly until all factors in the model had VIF values < 3 to exclude collinearity from the model. The model with the remaining explanatory factors made the basis for the RDA plot (Fig. 3).

## Supporting information


**Appendix** S1: Supporting Information
