# Supplementary material for: Effect of salinity on cable bacteria species composition and diversity
Source: Environ Microbiol. 2021 May 4;23(5):2605–16. doi: 10.1111/1462-2920.15484 (PMC8252435; doi:10.1111/1462-2920.15484)
Supplement: Supplementary file 1 — Appendix S1: Supporting Information [file EMI-23-2605-s001.doc]

**EFFECT OF SALINITY ON CABLE BACTERIA SPECIES COMPOSITION AND DIVERSITY**

Ann-Sofie Dam^1^, Ian P. G. Marshall^1^, Nils Risgaard-Petersen^1,2^, Laurine D. W. Burdorf^1,3^, and Ugo Marzocchi^1,4*^

^1^ Center for Electromicrobiology, Section for Microbiology, Department of Biology, Aarhus University, Aarhus, Denmark

^2^ Section of Aquatic Biology, Department of Biology, Aarhus University, Aarhus, Denmark.

^3^ Centre of Excellence for Microbial Systems Technology, Department of Biology, University of Antwerp, 2610 Wilrijk, Belgium

^4^ Center for Water Technology (WATEC), Department of Biology, Aarhus University, Aarhus, Denmark

**SUPPLEMENTARY INFORMATION**

**Table S1 – Spearmann correlation matrix**

**Table S2 - 16S rRNA global data sets**

**Table S3 - Isolation source of Desulfobulbaceae species in the Silva SSU database**

**Table S4 - Salinity categories in figure 5**

**Table S5-S8 - PCR programs**

**Table S9-S10 – Barcodes for laboratory incubations**

| rho | TOC | TOC/N | TN | Tsul | Salinity | Temperature | Pore water O_2_ |
| --- | --- | --- | --- | --- | --- | --- | --- |
| TOC | - |  |  |  |  |  |  |
| TOC/N | -0.16 | - |  |  |  |  |  |
| TN | 0.98 **** | -0.27 | - |  |  |  |  |
| Tsul | 0.86 **** | 0.00 | 0.83 **** | - |  |  |  |
| Salinity | 0.62 **** | 0.12 | 0.58 **** | 0.73 **** | - |  |  |
| Temperature | 0.53 **** | 0.44 *** | 0.45 *** | 0.70 **** | 0.51 **** | - |  |
| Pore water O_2_ | -0.19 | 0.87 **** | -0.27 | -0.09 | 0.19 | 0.40 | - |

**Table S1.** Spearman correlation test between the environmental parameters available from Klier et al. 2018, *i.e.*, Salinity, Temperature, Total organic carbon (TOC), total sulphur (Tsul), total nitrogen (TN), total organic carbon:nitrogen (TOC/N). No sign P > 0.05; * P ≤ 0.05; ** P ≤ 0.01; *** P ≤ 0.001; **** P ≤ 0.0001.

| **Site** | **Samples with cable bacteria** | **Site description** | **Salinity (‰)** | **Average % of CB per sample (± SD)** | **Average no of unique CB ASV (± SD)** | **Study / SRA code** |
| --- | --- | --- | --- | --- | --- | --- |
| **Delaware Bay, USA** | 1 | Estuary | 6 | 0.05 | 1 | Campbell et al 2013 |
| **Dianchi Lake, China** | 1 | Freshwater lake, eutrophic | 0.1 | 0.18 | 1 | Dai et al 2016 |
| **Yellow Creek, Ohio, USA** | 1 | Freshwater stream | 0.1 | 0.5 | 1 | SRP127620 |
| **Saltmarsh, Louisiana, USA** | 2 | Saltmarsh | 0.01 | 0.08-0.16 | 1 | Rietl et al 2016 |
| **Lake Svarttjärn, Sweden** | 6 | Oligotrophic, freshwater lake | 0.1 | 0.27 ± 0.13 | 1 | SRP035352 |
| **Lake Vallentuna, Sweden** | 11 | Eutrophic, freshwater lake | 0.1 | 0.17 ± 0.12 | 1 | SRP035352 |
| **Arctic deep sea: Haakon Mosby Mud Volcano** | 4 | Marine | 35 | 0.38 ± 0.35 | 1 | SRP042162 |
| **Havre-aux-Maisons lagoon, Canada** | 6 | Temperate, costal lagoon | 31 | 2.5 ± 1.9 | 1.0 ± 0.6 | SRP027405 |
| **East Arm, Darwin Harbour, Australia** | 15 | Tropical estuary | 2-41 | 0.25 ± 0.4 | 1.5 ± 1.1 | ERP022659 |
| **Shoal Bay, Buffalo Creek, Darwin Harbour, Australia** | 22 | Tropical estuary, hypereutrophic | 18-37 | 0.18 ± 0.3 | 1.3 ± 0.6 | ERP022659 |
| **Caspian Sea** | 1 | Brackish | 11 | 0.2 | 1 | Mahmoudi et al 2015 |
| **Les Salins du lion, France** | 4 | Brackish coastal bird reserve | 14-36 | 1.78 ± 1.79 | 1 | Aubé et al 2016 |
| **Berre l’Étang, France** | 6 | Hydrocarbon contaminated retention basin | 20-28 | 1 ± 1.8 | 3 ± 1.5 | Aubé et al 2016 |
| **Wells, Maine, USA** | 2 | Estuary | 30 | 0.12-0.07 | 1 | Rothenheber & Jones 2018 |
| **Bayou du Large, California, USA** | 1 | Freshwater wetland | 0.1 | 0.62 | 1 | Tremblay et al 2015 |
| **Sea of Bothnia, Baltic Sea** | 1 | Estuary | 6 | 0.10 | 1 | Klier et al 2016 |
| **Gotland Sea, Baltic Sea** | 21 | Estuary | 7-8 | 1.00 ± 1.85 | 3.5 ± 1.5 | Klier et al 2016 |
| **Belt Sea, Baltic Sea** | 21 | Estuary | 21 | 0.55 ± 0.40 | 2.1 ± 1.3 | Klier et al 2016 |
| **Skagerrak, Baltic Sea** | 5 | Marine | 35 | 0.71 ± 0.55 | 2.4 ± 1.5 | Klier et al 2016 |

**Table S2.** Information table on the 16S rRNA data sets collected from ncbi.nlm.nih.gov.

| **Genus** | **Species** | **Isolation source** | **Accession number** | **Reference** |
| --- | --- | --- | --- | --- |
| **Desulfobulbus** | *japonica* | Harbor sediment in Japan | AB110549 | Suzuki et al. 2007 |
| **Desulfobulbus** | *propionicus* | Freshwater mud in Germany | CP002364 | Pagani et al. 2011 |
| **Desulfobulbus** | *PR8_A05* | Estuarine sediment in France | HE600836 | Colin et al. 2013 |
| **Desulfubulbus** | Enrichment culture clone *MS_ACE_N4* | Marine calcareous sandy sediment, Mallorca | HQ400771 | Suárez-Suárez et al. 2011 |
| **Desulfobulbus** | *Sp. S4* | Anaerobic cellulolytic microbial consortium enriched from mangrove soil | JN688053 | Gao et al. 2014 |
| **Desulfobulbus** | *Sp. SR3* | Wastewater in Vietnam | KP059286 | Nguyen H. T., unpublished |
| **Desulfobulbus** | *Sp. SM41* | Marine sediment in the region of Skhira in the Gulf of Gabes, Tunisia | KU180232 | Kharret et al, unpublished |
| **Desulfobulbus** | *rhabdoformis* | Water-oil separation system, North Sea oil platform | U12253 | Lien et al. 1998 |
| **Desulfobulbus** | *Sp. BG25* | Salt marsh sediment | U85473 | Rooney Varga et al, unpublished |
| **Desulfocapsa** | *Sp. Cad626* | Lake Cadagno in Switzerland | AJ511275 | Peduzzi et al. 2003 |
| **Desulfocapsa** | *sulfexigens DSM 10523* | Marine | CP003985 | Finster et al, unpublished |
| **Desulfopila** | *aestuarii* | Harbor sediment in Japan | AB110542 | Suzuki et al. 2007 |
| **Desulfocapsa** | *Sp. La4.1* | Marine sediment, Nederlands | AF228119 | Wieringa et al., unpublished |
| **Desulfovibrio** | *Sp. G5V* | Tidal flat sediment, North Sea | AJ786069 | Koepke et al. 2005 |
| **Desulfoprunum** | *benzoelyticum* | Wastewater treatment plant, low NaCl | KJ766003 | Junghare & Schink, 2015 |
| **Desulforhopalus** | *Sp. LSv20* | Marine Arctic sediment | AF099057 | Sahm et al. 1999 |
| **Desulforhopalus** | *singaporensis* | Marine | AF118453 | Lie et al. 1999 |
| **Desulforhopalus** | *Sp. 16SWWS2-2-3* | Sediment of intertidal zone, Antarctica | KT266595 | Y. Han, unpublished |
| **Desulforhopalus** | *vacuolatus* | Temperate estuary | L42613 | Isaksen & Teske, 1996 |
| **Desulfotalea** | *arctica* | Marine Arctic sediment | AF099061 | Sahm et al. 1999 |
| **Desulfotalea** | *psychrophila* | Marine Arctic sediment | AF099062 | Sahm et al. 1999 |
| **Desulfotalea** | *Sp. SFA4* | Intertidal flat, North Sea | AJ318381 | Ruetters et al., unpublished |
| **Desulfurivibrio** | *alkaliphilus* | Sediment from highly alkaline soda lake, Egypt | CP001940 | Lucas et al., unpublished |
| **Desulfurivibrio** | *Sp. AMeS2* | Soda lake, Siberia | KF148062 | Sorokin D. Y., unpublished |
| **Dissulfuribacter** | *thermophilus* | Deep-sea hydrothermal vent |  | Slobodkin et al., 2013 |
| **Dissulfurimicrobium** | *hydrothermale* | hydrothermal pond of Uzon Caldera, Kamchatka ionic solute concentration: 2 mM | KT159733 | Slobodkin et al., unpublished |
| **Dissulfurirhabdus** | *thermomarina* | Shallow sea hydrothermal vent | KU051627 | Slobodkina et al., unpublished |
| **Desulfobulbus** | *Sp. IS6* | Marine sediment, Singapore | KF733438 | Enning D. R., unpublished |

**Table S3.** Isolation source of Desulfobulbaceae species in the Silva SSU database version 138.

| **Salinity category** | | **Salinity (psu)** |
| --- | --- | --- |
| **Freshwater**  F1  F2 | | 0.1 0.3 |
| **Brackish** | **Mesohaline**  M1  M2 | 6-8  14 |
|  | **Polyhaline**  P1  P2  P3  P4 | 18  20-21  25  28 |
| **Marine (Euhaline)**  E1  E2  E3  E4 | | 30-32  33-35  37-38  41 |

**Table S4.** Salinity categories in figure 5.

**PCR programs**

| Step | Time | Temperature |
| --- | --- | --- |
| **Initial heat activation** | 15 min. | 95°C |
| **3-step cycling** |  |  |
| Denaturation | 1 min | 94°C |
| Annealing | 1 min | 60°C |
| Extension | 1 min | 72°C |
| **Number of cycles** | 30 | |
| **Final extension** | 10 min | 72°C |

**Table S5.** PCR DNA contamination control (HotStarTaq® Master Mix Kit).

**16S Metagenomic Sequencing Library Preparation**

| Step | Time | Temperature |
| --- | --- | --- |
| **Reverse transcription** | 30 min | 50°C |
| **DNA polymerase activation** | 15 min | 95°C |
| **Thermal cycling** |  |  |
| Denaturation | 40 s | 94°C |
| Annealing | 45 s | 57°C |
| Extension | 1 min | 72°C |
| **Number of cycles** | 27 | |
| **Final extension** | 7 min | 72°C |
| **Chemistry** RT_PCR: Mastermix 5x OneStep Buffer RT-PCR kit (Qiagen, Copenhagen, Denmark) 10 µl, dNTP 2 µl (10 mM each). Forward Primer 3 µl, Reverse Primer 3 µl (primers at 10 pmol/µl), enzymemix 2 µl H_2_O 20 µl. | | |

**Table S6.** Reverse Transcriptase (RT)-PCR

| Step | Time | Temperature |
| --- | --- | --- |
| **DNA polymerase activation** | 3 min | 95°C |
| **Thermal cycling** |  |  |
| Denaturation | 40 s | 94°C |
| Annealing | 45 s | 55°C |
| Extension | 1 min | 72°C |
| **Number of cycles** | 10 | |
| **Final extension** | 7 min | 72°C |
| **Chemistry** Mastermix for adapter PCR: 2xKAPA HiFi Hotstart mix (Roche, Hvidovre, Denmark) 12.5 ul. Forward primer + adapter: 0.5 µl, Reverse primer; 0.5 µl (primers at 10 pmol/µl), template for each sample: 2 µl., H_2_O; 9.5 µl. | | |

**Table S7.** Adaptor PCR

| Step | Time | Temperature |
| --- | --- | --- |
| **DNA polymerase activation** | 3 min | 95°C |
| **Thermal cycling *** |  |  |
| Denaturation | 30 s | 95°C |
| Annealing | 30 s | 55°C |
| Extension | 30 s | 72°C |
| **Number of cycles** | 8 | |
| **Final extension** | 5 min | 72°C |
| **Chemistry** Mastermix: 2xKAPA, 12.5 µl index primer 1, 2.5 µl index primer 2, 2.5 µl template, 2.5 µl (primers at 10 pmol/µl), dH_2_O, 5.0 µl. | | |

**Table S8.** Index PCR

| Sediment Type | Treatment (Salinity) | Barcode |
| --- | --- | --- |
| Aarhus Harbor | 0.3 | N722, S506 |
| Aarhus Harbor | 21 | N723, S506 |
| Aarhus Harbor | 21 | N724, S506 |
| Brabrand Lake | 21 | N726, S506 |
| Brabrand Lake | 0.3 | N727, S506 |
| Aarhus Harbor | 0.3 | N728, S506 |
| Aarhus Harbor | 21 | N729, S506 |
| Aarhus Harbor | 0.3 | N716, S507 |
| Brabrand Lake | 21 | N718, S507 |
| Brabrand Lake | 21 | N719, S507 |
| Brabrand Lake | 0.3 | N720, S507 |
| Blank | - | N721, S507 |
| Blank | - | N722, S507 |

**Table S9**. Indices for Illumina sequencing for experiment 1.

| Sediment Type | Treatment (Salinity) | Barcode |
| --- | --- | --- |
| Brabrand Lake | 5 | N703, S518 |
| Brabrand Lake | 5 | N703, S520 |
| Brabrand Lake | 5 | N703, S521 |
| Brabrand Lake | 5 | N703, S522 |
| Brabrand Lake | 0.3 | N704, S513 |
| Brabrand Lake | 0.3 | N704, S515 |
| Brabrand Lake | 0.3 | N704, S516 |
| Brabrand Lake | 0.3 | N704, S517 |
| Brabrand Lake | 3 | N704, S518 |
| Brabrand Lake | 1.5 | N704, S520 |
| Brabrand Lake | 1.5 | N704, S521 |
| Brabrand Lake | 1.5 | N704, S522 |
| Brabrand Lake | 1.5 | N705, S513 |
| Brabrand Lake | 3 | N705, S515 |
| Brabrand Lake | 3 | N705, S516 |
| Brabrand Lake | 3 | N705, S517 |
| Blank | - | N705, S521 |
| Blank | - | N705, S522 |

**Table S10**. Indices for Illumina sequencing for experiment 2.
